# Genome Sequence Data of Lactobacillus ruminis ICIS-540, Isolated from the Intestine of a Healthy Woman

**DOI:** 10.1128/MRA.00900-20

**Published:** 2020-12-03

**Authors:** Elena V. Ivanova, Anastasia V. Bekpergenova, Natalia B. Perunova, Sergey V. Andryuschenko, Taisia A. Bondarenko, Oleg V. Bukharin

**Affiliations:** a Orenburg Federal Research Center of the Ural Branch of the Russian Academy of Sciences, Institute for Cellular and Intracellular Symbiosis, Orenburg, Russia; University of Maryland School of Medicine

## Abstract

Data on the draft genome sequence of Lactobacillus ruminis ICIS-540 are presented in this report. This *Lactobacillus* strain was isolated from the human colon as a prospective probiotic candidate. The genome size was 2,397,517 bp (G+C content, 42.7%). Annotation revealed 2,847 coding sequences, including 2,573 proteins.

## Abstract (precis)

Data on the draft genome sequence of Lactobacillus ruminis ICIS-540 are presented in this report. This *Lactobacillus* strain was isolated from the human colon as a prospective probiotic candidate. The genome size was 2,397,517 bp (G+C content of 42.7%). Annotation revealed 2,847 coding sequences, including 2,573 proteins.

## ANNOUNCEMENT

Lactobacilli are natural colonizers of the human gastrointestinal tract (GIT) (1). Lactobacilli belong to a very large and heterogeneous bacterial group with widely differing properties, natural niches, and metabolisms. Furthermore, different *Lactobacillus* species may interact with antigen-presenting cells and induce different cytokine patterns, leading to different immune effector pathway activation (2).

Lactobacilli are immunobiotics (рrobiotic microorganisms able to regulate the immune system) which are capable of interacting with the host’s immune and nonimmune cells and thereby impact mucosal and systemic immune responses (3–5).

Here, we present a draft genome sequence of Lactobacillus ruminis strain ICIS-540, isolated from a human feces sample from a healthy 38-year-old woman from Orenburg, Russia. The collection and sequencing of the isolate have approval from the institutional review board (IRB) of our institution. The sample was diluted in a 0.9% NaCl solution to 107-fold by mass and cultivated on a Schaedler agar plate (HiMedia Laboratories Pvt. Limited). The strain was stored in the laboratory culture collection from April 2014.

The taxonomy of this strain, previously identified as Lactobacillus acidophilus by matrix-assisted laser desorption ionization–time of flight (MALDI-TOF) mass spectrometry of cell proteins (Bruker Daltonics, Germany) with Biotyper software, was verified by average nucleotide identity (ANI) of genome sequences using the NCBI Prokaryotic Genome Annotation Pipeline (PGAP) version 4.8 (6) as described in references 7 and 8 and was reidentified as Lactobacillus ruminis. Genome sequences of strain ICIS-540 are 97.488% identical by ANI to the type genome of Lactobacillus ruminis, with 72.2% coverage of the genome.

Strain *L. ruminis* ICIS-540 was cultivated in 5 ml of Schaedler broth (HiMedia Laboratories Pvt. Limited) for 24 hours at 0.9% O_2_ and 9% CO_2_ atmosphere and temperature of 37°C in a CO_2_ incubator (Binder, Tuttlingen, Germany). The DNA extraction procedure was performed as described in our previous work (9). The obtained culture was centrifuged at 4,000 × *g* for 6 min, sediment was resuspended in 50 μl of Tris-buffered saline with 2 μg of lysozyme from chicken egg white, and the suspension was incubated at 37°C for 1 hour and then homogenized mechanically for 1 min by 1.4-mm silica beads at a 6.5 m/s speed. DNases were inactivated by heating the suspension to 95°C for 10 minutes; proteins were degraded by the addition of 2 μl of a 100 mg/ml proteinase K solution and 50 μl of a 10% SDS solution to the suspension with a subsequent incubation at 60°C for 60 min. The extracted DNA solution was purified by the phenol-chloroform method (10) and precipitated by ethanol (11). The centrifuged DNA precipitate was dissolved in 30 μl of Milli-Q deionized water.

The DNA library was prepared by the Nextera XT DNA sample preparation kit (Illumina, San Diego, CA). The genome library was sequenced by a MiSeq desktop sequencer in a 2 × 300-nucleotide run using the MiSeq reagent kit version 3 (Illumina). The 2,521,939 sequence reads were generated. The quality trimming of the reads was performed by the sliding window mode of the Trimmomatic program version 0.36 (12). The genome was assembled *de novo* by the SPAdes software (St. Petersburg genome assembler) version 3.10.1 (13). The genome assembly yielded 611 contigs covering 2,397,517 bp with an *N*_50_ value of 44,454 bp, a G+C content of 42.7%, and an average coverage of 35.7×. Contigs were additionally examined by the QUAST software version 4.3.

Annotation of the genome was performed by the NCBI PGAP version 4.8 (6). The contamination screen revealed the adaptor sequence contaminations in the ends of three contigs, namely, NODE_247, NODE_335, and NODE_559. The contaminations were removed from the final assembly. The annotated genome revealed 2,847 coding sequences, including 2,573 proteins, 164 pseudogenes, 26 rRNA genes, 80 tRNA genes, and 4 noncoding RNA genes.

Software was used with the default settings and parameters unless otherwise specified.

### Data availability.

The BioProject number for the sequenced strain is PRJNA529347. The Sequence Read Archive data for this project are available in the NCBI SRA under the number SRP191964. The whole-genome shotgun project has been deposited at DDBJ/ENA/GenBank under the accession number SRNR00000000. The version described in this paper is the first version, SRNR00000000.1.
